# Artificial intelligence in proliferative diabetic retinopathy: advancing diagnosis, precision surgery, and anti-VEGF therapy optimization

**DOI:** 10.3389/fmed.2025.1644456

**Published:** 2025-09-09

**Authors:** Geng-Qian Ke, Yang-Jun Fu, Zhuo-Han Huang, Shi-Xue Dai, Yun-Hua Wen, Hai-Xiang Lv

**Affiliations:** ^1^The First School of Clinical Medicine, Southern Medical University, Guangzhou, Guangdong, China; ^2^Department of Ultrasound, Guangdong Provincial People’s Hospital Ganzhou Hospital, Ganzhou Municipal Hospital, Ganzhou, Jiangxi, China; ^3^The First School of Clinical Medicine, Fuzhou Medical College of Nanchang University, Fuzhou, Jiangxi, China; ^4^Department of Gastroenterology, Guangdong Provincial Geriatrics Institute, National Key Clinical Specialty, Guangdong Provincial People’s Hospital (Guangdong Academy of Medical Sciences), Southern Medical University, Guangzhou, Guangdong, China; ^5^Department of Gastroenterology, Geriatric Center, National Regional Medical Center, Guangdong Provincial People’s Hospital Ganzhou Hospital, Ganzhou Municipal Hospital, Ganzhou, Jiangxi, China; ^6^Department of Ultrasound, Sanyuanli Street Community Health Service Center, Baiyun District, Guangzhou, Guangdong, China

**Keywords:** anti-VEGF therapy, deep learning, machine learning, proliferative diabetic retinopathy, artificial intelligence

## Abstract

Proliferative diabetic retinopathy (PDR) represents the most advanced and vision-threatening stage of diabetic retinopathy (DR) and remains a leading cause of blindness in individuals with diabetes. This review presents a comprehensive overview of recent advances in the application of artificial intelligence (AI) for the diagnosis and treatment of PDR, emphasizing its clinical potential and associated challenges. The role of vascular endothelial growth factor (VEGF) in the pathogenesis of PDR has become increasingly clear, and AI offers novel capabilities in retinal image analysis, disease progression prediction, and treatment decision-making. These advancements have notably improved diagnostic accuracy and efficiency. Furthermore, AI-based models show promise in optimizing anti-VEGF therapy by enhancing therapeutic outcomes while reducing unnecessary healthcare expenditures. Future research should focus on the safe, effective, and ethical integration of AI into clinical workflows. Overcoming practical deployment barriers will require interdisciplinary collaboration among technology developers, clinicians, and regulatory bodies. The strategies and frameworks discussed in this review are expected to provide a foundation for future AI research and clinical translation in fields of PDR.

## 1 Introduction

Diabetic retinopathy (DR), a microvascular complication of diabetes, has become an escalating global health concern. As the number of diabetic patients rises, so also does the burden of DR-related vision loss. Persistent hyperglycemia damages retinal blood vessels; in advanced stages, this can lead to vitreous hemorrhage, retinal detachment, and ultimately, irreversible blindness (1).

Proliferative diabetic retinopathy (PDR) represents the most vision-threatening stage of DR. Its pathogenesis is closely associated with elevated levels of VEGF, a key molecule promoting pathological angiogenesis (2), increased vascular permeability (3), and heightened inflammatory responses within the retinal microenvironment (4). VEGF expression is further upregulated under oxidative stress and hypoxic conditions—both common in diabetes—driving progressive neovascular complications. Accumulating evidence suggests that vitreous VEGF levels strongly correlate with oxidative stress markers, reinforcing its central role in disease progression (5).

Despite significant advances in understanding DR pathophysiology and the widespread use of anti-VEGF therapies—particularly for PDR and diabetic macular edema (DME) (6)—several critical clinical challenges persist. Notably, DR diagnosis still largely depends on the manual interpretation of fundus photographs by ophthalmologists, a process that is time-consuming and resource-intensive (7). In response, AI—specifically deep learning and large language models (LLMs)—has emerged as a transformative tool. Recent studies demonstrate that AI systems can accurately and reliably detect various stages of DR, including PDR, with the potential to alleviate diagnostic workload and improve early detection (8).

Beyond diagnosis, AI is increasingly being explored for its potential to assist in therapeutic decision-making and postoperative monitoring. For instance, integrating AI into anti-VEGF therapy workflows may allow for more personalized and predictive PDR management. Given the pathological overlap between PDR and DME (9, 10), this AI-guided strategy could have wide-reaching clinical implications. While DME frequently co-occurs with PDR, this review focuses exclusively on AI applications specific to PDR.

This narrative review aims to provide a comprehensive overview of recent advances in AI applications for PDR, encompassing automated diagnosis, individualized treatment planning, and follow-up evaluation. By highlighting AI’s potential to enhance clinical decision-making, improve workflow efficiency, and optimize patient outcomes, this article serves as a practical resource for retinal specialists and ophthalmic surgeons managing the complexities of PDR.

## 2 Methods

This narrative review was conducted to present a method that synthesizes recent developments in the application of AI in the diagnosis, surgical management, and anti-VEGF therapy optimization for PDR. Relevant literature was identified through systematic searches of PubMed, Web of Science, Embase, and Google Scholar databases. The search period was from January 2015 to June 2025. The following keywords were used in various combinations: “proliferative diabetic retinopathy,” “PDR,” “artificial intelligence,” “deep learning,” “machine learning,” “anti-VEGF therapy,” “retinal image analysis,” and “precision surgery”.

Inclusion criteria of this review included: (1) peer-reviewed articles or conference papers; (2) studies focused on AI applications in PDR or DR-related diagnosis/treatment; (3) articles describing clinical studies, model development, or review of AI in DR. Exclusion criteria included: (1) non-English publications, (2) case reports or letters without sufficient methodology, and (3) studies focused solely on non-proliferative DR or other unrelated retinal diseases.

A total of over 100 articles were included based on their relevance, novelty, and contribution to the understanding or advancement of AI applications in PDR management (Table 1).

**Table 1:** Classification of Diabetic Retinopathy and AI Applications.

Classification method	Classification basis	Classification criteria	AI applications and breakthroughs
International Clinical Classification	Severity of lesion	No Retinopathy (0): No significant signs of retinopathy. Mild NPDR (1): Presence of microaneurysms only. Moderate NPDR (2): Retinal hemorrhages, hard exudates, or cotton wool spots not meeting severe NPDR criteria. Severe NPDR (3): Microvascular abnormalities, numerous hemorrhages, intraretinal/preretinal hemorrhages, or venous beading. PDR (4): Neovascularization or fibrous membranes on the retina or optic disk.	Researchers developed a hybrid deep learning model (E-DenseNet) integrating EyeNet and DenseNet to improve DR lesion classification accuracy (29).
ETDRS classification	Lesion characteristics and severity	Mild NPDR: Characterized by microaneurysms. Moderate NPDR: Presence of retinal hemorrhages, hard exudates, and other lesions. Severe NPDR: Intraretinal microvascular abnormalities. PDR: Defined by the presence of neovascularization.	Researchers proposed an AI technique (ABiD) using forward-backward compensation to improve classification near decision boundaries (82).
American Academy of Ophthalmology Classification	Risk of lesion progression and characterization	Normal or Mild NPDR: Normal fundus or few microaneurysms. Mild to Moderate NPDR: Microaneurysms with few hemorrhages or hard exudates. Severe NPDR & Non-High-Risk PDR: Advanced lesions without high-risk features. High-Risk PDR: Meets ≥3 of 4 criteria: neovascularization, optic disk involvement, extensive neovascularization, or vitreous/peripheral hemorrhage.	Researchers applied a Mask R-CNN model to quantify microaneurysms and proposed a ResNeXt-based algorithm (ADRPPA) to assess DR severity over time (83).

Diagrams illustrating the associations investigated in this study are provided below: NPDR, Nonproliferative Diabetic Retinopathy; PDR, Proliferative Diabetic Retinopathy; DR, Diabetic Retinopathy; ABiD, Asymmetric Bi-Classifier Discrepancy Minimization.

## 3 Advances in AI for PDR diagnosis

DR, a leading cause of blindness in individuals with diabetes, results in progressive and often irreversible visual impairment (11). Recent advances in AI technologies have enabled large-scale screening and the development of tailored predictive models, thereby improving the efficiency and cost-effectiveness of DR screening programs (12). Modern ophthalmic computer-aided diagnosis (CAD) systems demonstrate low diagnostic error rates and offer significant savings in time, cost, and clinical manpower compared to traditional DR screening methods (13). Machine learning (ML) algorithms can effectively detect, localize, and quantify pathological features of DR, mimicking the human brain’s pattern recognition capabilities. Furthermore, by leveraging patterns autonomously identified through unsupervised convolutional neural networks (CNNs), these algorithms can accurately classify the various stages of DR (14).

### 3.1 Deep learning-based image learning

Arora et al. employed convolutional layering and compound scaling strategies in AI models to develop the EfficientNet framework (15). Specifically, the EfficientNetB0 model was optimized in terms of depth, width, and resolution to classify DR severity (16). Recent studies have utilized Generative Adversarial Networks (GANs) to generate synthetic fundus images, thereby enriching the diversity of training datasets. These synthetic images were incorporated into DiaGAN-CNN, a transfer learning-based deep learning model, to improve the accuracy of image-based DR classification (16). The final model achieved an accuracy of 0.84255, a quadratic weighted Kappa of 0.79565, and an AUC exceeding 0.90 (17).

Chao Chen et al. adopted Inception-V3 as the base architecture, reduced network complexity using Global Average Pooling (GAP), and integrated a fully connected layer with ReLU activation and a Softmax output layer. The model was trained on the ImageNet and Kaggle DR datasets, and the system was optimized through asynchronous calling mechanisms, multithreading, and microservice architecture (18). Using the Baidu EasyDL platform, Cao et al. applied a transfer learning approach, retraining the upper-layer network parameters to construct a new classifier based on a pre-trained model and a publicly available diabetic fundus disease dataset from Kaggle. Their model achieved a Kappa coefficient of 1.00 (19).

Collectively, these technical advancements have substantially improved the accuracy, consistency, and clinical applicability of AI-based diagnostic tools for DR and PDR detection.

### 3.2 Diversity input and overfitting prevention for data analysis

Researchers have noted that limited demographic diversity in training datasets may compromise the generalizability of AI models, resulting in reduced accuracy when applied to broader or underrepresented populations. To mitigate this issue, large-scale public datasets containing DR images of varying severity levels are commonly used to ensure adequate variability (15). In addition, a two-stage training strategy was employed in conjunction with the NASNet-Large pre-trained model to address the risk of overfitting due to excessive oversampling. This approach incorporated decision tree algorithms and the Synthetic Minority Over-sampling Technique (SMOTE) to manage data imbalance and improve model generalization (20).

### 3.3 Clinical validation of AI models

A dataset of retinal scans from Brazilian patients was used to develop and validate a deep learning algorithm capable of diagnosing various stages of DR, including PDR. The algorithm achieved a sensitivity of 97.8%, a specificity of 61.4%, and an area under the ROC curve of 0.89, highlighting its high sensitivity and overall diagnostic performance (21).

Beyond imaging, Fatma et al. proposed a hybrid model that integrates metabolomics data to enhance both the interpretability and predictive power of DR diagnosis. Model performance was validated using multiple metrics, including 10-fold cross-validation and SHAP (SHapley Additive exPlanations), demonstrating improved predictive accuracy and greater transparency in clinical interpretation (22).

To further improve generalizability across imaging devices, Zhang et al. introduced a novel preprocessing method, Single-Channel Standard Deviation Normalization (SCSDN). SCSDN maintained consistent performance across images acquired from various fundus camera models, minimized algorithm dependency on specific hardware, reduced clinical deployment costs, and significantly improved diagnostic accuracy in real-world DR validation scenarios (23).

Collectively, these clinical validations underscore the robustness, adaptability, and increasing reliability of AI-based diagnostic systems in detecting and stratifying PDR across diverse clinical settings (Table 2).

**Table 2:** Comparison of model performance in PDR and DME.

Category	AI model	Dataset	Task	Performance Metrics	Limitations
Diagnosis	E-DenseNet (29) (2022)	EyePACS, APTOS 2019, MESSIDOR, IDRiD	Grading diagnosis of Diabetic Retinopathy (DR)	ACC: Average 91.2% SEN: Average 96% SPE: Average 69% DSC: Average 92.45% QKS: Average 0.883	1. Relatively low specificity (average 69%). 2. Suboptimal capability in identifying PDR, with AUC only 76%.
	ABiD (82) (2024)	One toy dataset (inter-twining moons), two public datasets (IDRiD, DDR) and one private dataset (Aier)	Addressing Grade Shift Domain Adaptation (GSDA) problem	ABiD (ABiD‡) method showed overall Acc. improvements of 7.44% (8.42%), 2.45% (5.05%), 3.67% (4.52%) and 3.00% (12.88%), 6.19% (9.25%), 3.06% (3.61%) respectively.	1. Domain adaptation process requires model training, demanding substantial computational resources. 2. Image-level supervised training poses challenges for accurate lesion detection.
	ADRPPA (83) (2024)	EyePACS dataset	Prediction of Diabetic Retinopathy progression	Recall, precision, and F1-score were 0.338 (95% CI: 0.228–0.451), 0.561 (95% CI: 0.405–0.714), and 0.422 (95% CI: 0.299–0.532), respectively.	The time intervals between the encounters varied significantly Other known RDR features beyond microaneurysms were not explored. Ensemble of multiple CNNs or inclusion of multimodal data to enhance prediction accuracy and reliability was not investigated.
	EfficientNetB0 (15) (2024)	Kaggle 46	Classification of Diabetic Retinopathy severity	Maximum accuracy: 97.1%	Potential bias introduced by the dataset. Lack of enrichment for representation of minority classes
	Hybrid Explainable Artificial Intelligence Models (22) (2024)	T2DM dataset	DR grading prediction and metabolic biomarker discovery	SVC + RF: Accuracy: 86.11%, Precision: 83.39%, F1-Score: 84.38% SVC + DT: Accuracy: 85.80%, Precision: 83.48%, F1-Score: 84.75% SVC + LR: Accuracy: 83.91%, Precision: 80.79%, F1-Score: 81.41% SVC + MLP: Accuracy: 89.58%, Precision: 87.18%, F1-Score: 88.20%	1. Lack of extensive clinical validation. 2. Class imbalance.
	IDx-DR (84) (2023)	Retinal image dataset from Polish diabetic clinics	Automated screening for RDR	Sensitivity: 99%, Specificity: 68% (For RDR); Sensitivity: 99%, Specificity: 44% (For any DR)	1. Sample selection bias. 2. Insufficient external generalizability.
	Medios AI (84) (2023)	Retinal image dataset from Polish diabetic clinics	Automated screening for RDR	Sensitivity: 95%, Specificity: 80% (For RDR); Sensitivity: 89%, Specificity: 90% (For any DR)	1. Sample selection bias. 2. Insufficient external generalizability.
	Deep learning models (U-Net et al.) (85) (2023)	Public Kaggle EyePACS dataset	Five-stage classification of DR	Accuracy ranges from 82.00 to 97.92%	1. Generalization capability requires validation. 2. High computational cost.
	DeepDR (86) (2023)	Consecutive T2DM patients referred to a tertiary specialist diabetes eye clinic (Sep-Dec 2019)	Screening and grading of DR	Compared to clinical examination: AUC: 0.921, Sensitivity: 89.1%, Specificity: 100%, PPV: 100%, NPV: 91.4%, DE: 94.9% Compared to the standard fundus camera: AUC:0.883, Sensitivity: 83.2%, Specificity: 100%, PPV: 100%, NPV: 87.3%, DE: 92.2%	1. Lack of integration with different imaging modalities or sources to validate method versatility. 2. Dependence on image quality.
Surgery	Coarse-to-fine DR Network, CF-DRNet (25) (2020)	Public IDRiD and Kaggle fundus image datasets	Preoperative grading of DR severity	CF-DRNet achieved an accuracy of 60.20%, sensitivity of 69.61%, and specificity of 88.78%.	1. Requires designing a finer network to reduce confusion between the 4 stages of DR severity.
	Semantic Segmentation Convolutional Neural Network (27) (2021)	Ophthalmology Department, Health Campus, Universiti Sains Malaysia	Intraoperative neovascularization detection and localization in Proliferative Diabetic Retinopathy (PDR)	Average specificity was 0.9976, indicating 99.76% of Not-Neo pixels were correctly classified.	1. Insufficient data scale. 2. Risk of missing fine vessels.
	Ensembled U-Net Architecture (Ensembled U-Nets) (33) (2024)	OCTA scan dataset	Intraoperative microaneurysm segmentation in DR	Dice loss model performed best on the DCP layer (F1 = 0.67); ensemble strategy improved recall.	1. Limited data scale. 2. Risk of missing tiny MAs.
	XGBoost-based Ensemble (39) (2025)	Preoperative and intraoperative routine care EHR data from patients	Postoperative infection early prediction and clinical decision support	Calibration slope: 0.85–0.95 (close to ideal value of 1) Calibration intercept: −0.02 to −0.13 (close to ideal value of 0)	1. Absence of sensitive variables. 2. Data imbalance issue
Treatment	AlphaFold 3 (AF3) (47) (2024)	Training data: Protein Data Bank (PDB), sequence databases, nucleic acid data	Prediction of complex structures involving proteins, nucleic acids, small molecules, ions, and modified residues	Comprehensive enhancement in joint prediction performance for multiple types of biomolecules.	1. Stereochemical errors. 2. Hallucination issues.
	CADNet(Convolutional Attention-to-DME Network) (42) (2020)	127 subjects receiving three consecutive anti-VEGF injections	Prediction of anti-VEGF treatment response	Average AUC was 0.866; average precision, sensitivity, and specificity were 85.5, 80.1, and 85.0%, respectively.	1. Small sample size. 2. Failure to differentiate between anti-VEGF drugs.
	Xception-MLP Hybrid Architecture (49) (2024)	272 anti-VEGF-treated DME eyes	Prediction of clinical metrics post anti-VEGF treatment	Xception-MLP significantly outperformed pure CNN.	1. Deficiencies in retrospective data. 2. Lack of external validation.

Diagrams illustrating the associations investigated in this study are provided below: (A) Data description: The year in the table is the time of publication of the literature related to the model. There are differences and diversity in the performance metrics of AI reported in different literatures; Hybrid Explainable Artificial Intelligence Models (SVC + RF etc.) represent the joint application of multiple algorithms; (B) RDR, Referable Diabetic Retinopathy; DR, Diabetic Retinopathy; AUC, Under the Curves; PPV, Positive Predictive Value; NPV, Negative Predictive Value; DE, Diagnostic Effectiveness; SVC, Support Vector Machines; RF, Random Forest; DT, Decision Tree; LR, Logistic Regression; MLP, Multilayer Perceptrons; F1-Score, The Harmonic mean of Precision and Recall Values; ACC, Average Accuracy; SEN, Specificity; DSC, Dice similarity coefficient; QKS, The quadratic Kappa score; GSDA, Gradeskewed Domain Adaptation.

## 4 The value of AI in guiding PDR precision surgery

As AI technology advances, its use in the diagnosis, treatment planning, and postoperative evaluation of PDR becomes more ubiquitous.

### 4.1 Preoperative assessment

#### 4.1.1 CNNs-based innovations

Accurate preoperative evaluation is essential for ensuring surgical success. In recent years, CNN-based models have been extensively applied to screen and classify DR (24). These automated systems can distinguish between different pathological grades using fundus images or optical coherence tomography (OCT), thereby providing a scientific foundation for designing personalized surgical plans.

The hierarchical coarse-to-fine classification network (CF-DRNet), proposed by Wu et al., facilitates this process through a cascaded structure comprising a coarse classification step (No DR vs. DR) followed by fine-grained classification (four-stage DR grading). By incorporating an attention mechanism to enhance lesion feature extraction, the model achieved accuracies of 56.19 and 83.10% on the IDRiD and Kaggle datasets, respectively. This approach effectively addresses challenges such as inter-class similarity and data imbalance in DR classification (25).

In another study, accurate DR staging was achieved by integrating ultra-widefield fundus imaging (Optos) and OCT angiography (OCTA) into a deep convolutional neural network (DCNN). This multimodal framework achieved an area under the curve (AUC) of 0.964 and a specificity of 96.4% in differentiating between no evident DR (NDR) and proliferative diabetic retinopathy (PDR), demonstrating strong clinical utility, particularly in complex cases (26).

Tang et al. developed a semantic segmentation CNN that achieved 99.48% accuracy and an 84.66% Dice similarity coefficient in pixel-level detection of neovascularization. Its ability to localize lesion sites significantly outperformed traditional patch-based classification methods (27). Furthermore, Aleksandra et al. compared the performance of a standalone AI model with conventional techniques for early-stage DR staging. Their findings indicated that the AI model demonstrated higher sensitivity and specificity in detecting early lesions, enabling earlier identification of potential complications and formulation of individualized surgical plans. This study highlights how AI accelerates diagnostic workflows and enhances the detection of subtle pathological changes, which is essential for early clinical intervention (28).

#### 4.1.2 Hybrid architectures and attention mechanisms

Hybrid methods and attention-based models have further improved grading accuracy for PDR. Abdel et al. proposed a hybrid deep learning framework, E-DenseNet, which integrates a pre-trained EyeNet with the DenseNet architecture. This model achieved an 84% classification accuracy for PDR on the APTOS 2019 dataset. Its advantages include enhanced feature reuse through dense connectivity modules and strong robustness in cross-dataset validation (29).

Gu et al. combined a Vision Transformer (ViT) with class-specific residual attention (CSRA). The ViT module captured fine-grained pathological variations, while CSRA enhanced inter-class discriminability. The model achieved a PDR classification AUC of 0.9081 on the DDR dataset (30). Similarly, Mondal et al. introduced EDLDR, an ensemble model combining DenseNet101 and ResNeXt. With data augmentation using a GAN, it achieved an accuracy of 86.08% for five-class classification on the APTOS 2019 dataset. Grad-CAM visualization confirmed that the model accurately focused on PDR lesion regions (31).

### 4.2 Real-time surgical guidance systems

Accurate preoperative planning enabled by AI can be seamlessly integrated with real-time intraoperative guidance systems to enhance surgical outcomes. Building on diagnostic advancements, AI plays a pivotal role during surgery by leveraging detailed structural and flow information derived from non-invasive imaging modalities such as OCT and OCT angiography (32). The incorporation of AI into surgical workflows has markedly improved the precision of PDR treatment.

For example, U-Net-based ensemble models have been employed for the non-invasive segmentation of microaneurysms (33). Other deep learning algorithms applied to OCTA not only achieve high diagnostic accuracy for DR and referable status but also generate class activation maps (CAMs), which visualize specific pathological regions—such as foveal avascular zone (FAZ) alterations and vessel density changes—thus potentially guiding intraoperative decision-making directly on OCTA images (34). Additionally, AI-driven systems can distinguish pathological neovascularization (NV) from compensatory angiogenesis, enabling targeted interventions such as suppression of NV using high-intensity, low-intensity pulsed ultrasound (LIPUS; 0.5 MHz, 210 mW/cm (2)), inducing endothelial apoptosis via the p38 MAPK/ER stress signaling pathway (35). Accurate AI-aided segmentation of these features is essential for precise localization and surgical planning (32). As a result, real-time intraoperative imaging integration becomes feasible, significantly improving surgical precision.

PDR pathogenesis involves retinal microvascular occlusion, which promotes NV formation. These newly formed, fragile vessels are prone to rupture, frequently leading to vitreous hemorrhage and pathological foci (27). To address this, AI models are instrumental not only in anatomical localization but also in assessing neovascular activity. For instance, deep learning methods applied to ultra-widefield fluorescein angiography (UWF-FA) can detect neovascular leakage with high accuracy (AUC = 0.96), effectively distinguishing active lesions requiring treatment from confounding retinal features, thereby guiding laser or surgical planning (36). In ischemic regions, AI-guided application of low-intensity LIPUS (1.5 MHz, 30 mW/cm^2^) can promote AKT-mediated angiogenesis, enhance local vascular density, and support metabolic recovery (37). Michael et al. developed a deep learning algorithm capable of identifying NV in fundus images and providing real-time intraoperative feedback, thereby supporting targeted and adaptive interventions during surgery (27). Collectively, these AI-assisted technologies substantially enhance the safety and precision of PDR surgery.

### 4.3 Post-operative risk prediction models

AI also plays a pivotal role in postoperative management by enabling predictive analytics and personalized medicine strategies for patients with DR. For instance, few-shot learning (FSL) combined with explainable AI (XAI) has been used to quantify macular features from OCTA images, enabling accurate assessment of recovery trajectories and facilitating individualized treatment planning (38).

In another study, Siri et al. developed an XGBoost-based predictive model using electronic health record (EHR) data to estimate 7-day and 30-day postoperative infection risks, thereby supporting systematic monitoring and early clinical intervention (39). These AI-powered tools contribute to optimizing recovery protocols and reducing complication rates, ultimately enhancing long-term clinical outcomes in DR management.

## 5 The role of AI in guiding precision VEGF therapy

Research on the application of AI in anti-VEGF therapy specifically for PDR remains limited, largely due to the shared pathological basis between DME and PDR. This section focuses on the use of AI in anti-VEGF therapy for DME, with the aim of providing a reference for its potential application in PDR and facilitating future advancements in this field.

### 5.1 Current limitations and pathologic rationale

Current AI studies specifically targeting anti-VEGF therapy in PDR remain scarce, necessitating extrapolation from DME evidence. Research on AI applications targeting anti-VEGF therapy for PDR remains limited—a gap that underscores the need for a translational research paradigm informed by findings from DME. This approach is justified by three core pathological commonalities:

First, both DME and PDR share a VEGF-driven pathogenic mechanism. The pathogenesis of these conditions is closely associated with elevated VEGF levels (5, 40). Notably, oxidative stress under hypoxic conditions is significantly and positively correlated with VEGF concentrations in the vitreous fluid of patients with DME and PDR (41, 42). VEGF activates several downstream signaling pathways, including PLCγ–PKC–MAPK, PI3K–AKT, and RAC, which collectively regulate angiogenesis (43). While anti-VEGF therapy is the first-line treatment for DME (10), it also serves as an alternative or adjunctive option in PDR, with demonstrated therapeutic efficacy (44). Furthermore, VEGF forms complexes with proteins such as copper transport protein 1 (CTR1) via disulfide bonds (45), and assembles ternary structures involving endoglin (ENG), neuropilin 1 (NRP1), and VEGFR2 to enhance pro-angiogenic signaling (46). These pathogenic complexes can now be structurally analyzed using AI-based tools such as AlphaFold 3 (AF3), which predicts interactions among proteins, nucleic acids, small molecules, ions, and modified residues, providing novel insights into anti-VEGF therapeutic mechanisms (47).

Second, both diseases exhibit common imaging biomarkers of vascular mobility. DME is primarily induced by ischemia, which increases retinal capillary permeability and promotes microaneurysm formation (48). The resistance index (RI = [PSV – EDV] / PSV), an indicator of distal microvascular resistance, has been significantly associated with progression toward retinal non-perfusion. The vasculopathic processes seen in DME are expected to induce RI-like changes in vascular parameters in PDR as well (49). Wang et al. reported that diabetic patients with proliferative retinopathy exhibited the highest mean RI value (0.83), compared to significantly lower values in healthy controls (0.54, *p* ≤ 0.001) (50).

Third, there is a generalizability of treatment response prediction. Gross et al. demonstrated that anti-VEGF agents such as ranibizumab are effective in both PDR and DME, reducing the risk of visual field loss associated with panretinal photocoagulation (PRP) (51). Li et al. proposed a multimodal fusion architecture that separately extracts and hierarchically integrates structural and blood flow information, enhances feature representation via multiscale feature interaction, and applies a weighted average (Avg) of OCTA grading outcomes to avoid alignment-dependent feature fusion. The model uses ResNet50 as the backbone, optimized with the Adam optimizer and supported by data augmentation techniques. This multimodal fusion architecture demonstrates applicability to both PDR and DME (52).

### 5.2 AI can predict treatment outcomes

Understanding these molecular interactions enables AI to predict therapeutic responses, as demonstrated in the following predictive models. Medical AI, particularly in predicting patient responses to treatment, has demonstrated considerable promise—especially in forecasting outcomes of anti-VEGF therapy for DME. Deep learning models—particularly those based on OCT image analysis—have been extensively employed to evaluate initial patient responses to therapy.

In one study, Rasti et al. employed a deep learning algorithm to analyze OCT images obtained before and after treatment to assess the therapeutic response in DME patients undergoing anti-VEGF therapy. Performance metrics such as AUC, sensitivity, precision, and specificity were used to evaluate the predictive model (53).

To accurately predict best-corrected visual acuity (BCVA), central subfield thickness (CST), cube volume (CV), and cube average thickness (CAT) from multimodal data in DME patients receiving anti-VEGF therapy, Leng et al. developed a deep learning model integrating a convolutional neural network (CNN) and a multilayer perceptron (MLP) (54).

Alternatively, generative adversarial networks (GANs) have been employed to predict OCT image outcomes. These models effectively identified key biomarkers—including intraretinal fluid (IRF), subretinal fluid (SRF), and hard exudates (HE)—enabling more detailed forecasts of treatment response in DME patients. Such approaches further assist clinicians in predicting both short- and long-term therapeutic outcomes (55).

Xin et al. proposed a separate prediction model, demonstrating that cube-measured foveal volume (CMFV) provided more accurate estimates of initial anti-VEGF treatment efficacy than CST. Their deep learning model estimated CMFV from OCT images, incorporating additional differentiation techniques to enhance predictive performance (56).

In another study, Sastry et al. developed the Notal OCT Analyzer (NOA), a machine learning system for quantifying retinal fluid volumes—including SRF, IRF, and total retinal fluid (TRF)—which are critical for evaluating patient responses to anti-VEGF therapy. Their results showed that fluctuations in these volumes were associated with treatment efficacy, providing clinicians with improved insight into therapeutic outcomes and prognostic potential (57).

Building upon these foundational studies, our analysis proposes a unified framework to optimize the role of AI in personalized anti-VEGF therapy.

### 5.3 AI can optimize treatment plans

China bears a substantial burden of diabetic retinopathy (DR), and AI-assisted solutions can be particularly valuable in economically underdeveloped regions. These approaches have the potential to significantly reduce the workload associated with image grading and lower capital expenditures (58).

To enhance treatment planning and minimize the unnecessary use of high-cost drugs, Anwesa et al. developed a hybrid deep learning model to predict responses to anti-VEGF therapy in patients with DME (59). Ruijie et al. applied a machine learning regression model trained on real-world data to predict the short-term efficacy of anti-VEGF therapy in DME patients. Model performance was assessed using mean absolute error (MAE), mean squared error (MSE), and the coefficient of determination (R^2^). This predictive capability is essential for both clinical and economic decision-making related to the short-term outcomes of anti-VEGF therapy (60).

AI-based prediction of anti-VEGF treatment response in DR and DME supports the development of personalized and effective therapeutic strategies (61).

### 5.4 AI can aid clinical decision-making and improve treatment efficiency

In clinical decision-making, AI provides predictive insights to assess responses to anti-VEGF therapy, mitigate associated risks, and optimize therapeutic outcomes. Ying et al. developed a machine learning model to predict changes in best-corrected visual acuity (VA) in patients with DME 1 month after anti-VEGF treatment. This model supports clinicians in making informed treatment decisions, tailoring individualized therapeutic strategies, and managing patient expectations regarding treatment outcomes (62).

To obtain a more objective evaluation of visual function, researchers have used OCT imaging to infer visual acuity in DME patients (63). The resulting predictive models achieved R^2^ values of 99.9% for DR, 97.7% for early DR, 93.9% for DME, and 98.4% for strong responders in the training set, and 96.3, 96.8, 79.9, and 96.3%, respectively, in the validation set.

Yuhui et al. combined multi-omics analysis with machine learning to enable early diagnosis of DR and DME and to predict responses to anti-VEGF therapy. Their model accurately forecasted early DR progression and treatment response in DME patients, offering a novel tool for clinical diagnosis and therapeutic planning (64).

**Figure 1:**
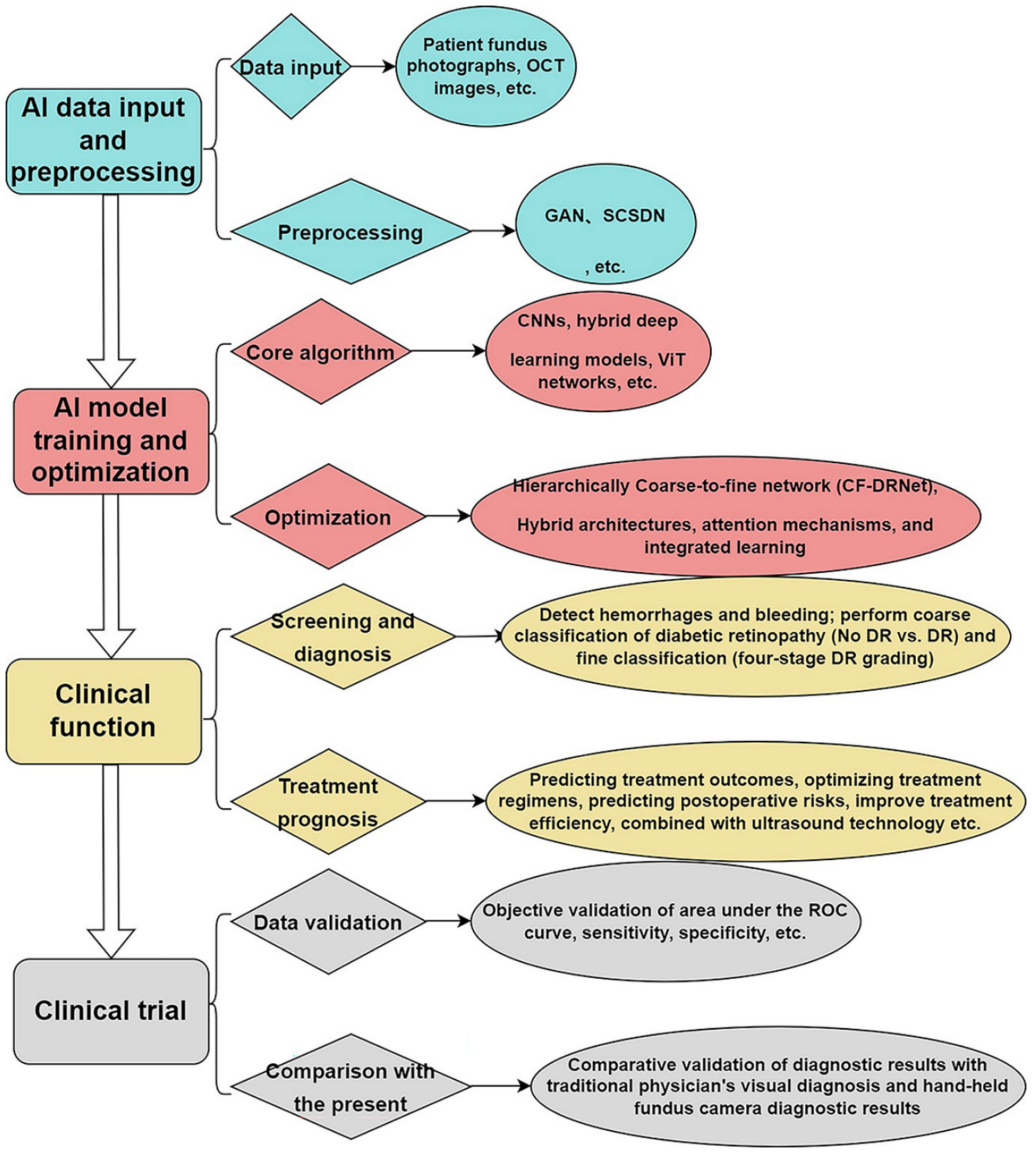
AI-driven model for PDR diagnosis and treatment.

Soumya et al. integrated AI algorithms to automate OCT data segmentation and analysis, resulting in reduced processing time and cost, faster diagnostic reporting, and improved workflow efficiency (65) (Figure 1).

Diagrams illustrating the associations investigated in this study are provided below: (A) Fundus photographs and OCT images are input and preprocessed using GAN and SCSDN to enhance image quality and improve model accuracy. (B) The core algorithm leverages CNNs, hybrid deep learning models, and ViT networks, incorporating optimization steps to enhance model performance, classification accuracy, and generalization. (C) The AI model detects lesion features in fundus images and classifies PDR, providing clinicians with a reliable diagnostic basis while enhancing treatment efficiency and patient quality of life. (D) Data Validation: The model’s performance was evaluated by calculating the area under the ROC curve, sensitivity, specificity, and other metrics. Its accuracy and feasibility were verified by comparing it with traditional physicians and handheld fundus cameras. (E) OCT, Optical Coherence Tomography; GAN, Generative Adversarial Network; SCSDN, Single Channel Standard Deviation Normalization; CNN, Convolutional Neural Network; ViT networks, Vision Transformer networks; ROC Curve, Receiver Operating Characteristic Curve.

## 6 Critical appraisal: technological divide and breakthrough paths for AI in PDR management

### 6.1 Inherent limitations of AI models applied to PDR

One major limitation of current AI models is their heavy reliance on training data from limited geographic regions, resulting in notable generalization issues. For instance, Ting et al. developed a deep learning system (DLS) for diabetic retinopathy screening using multi-country datasets, with the AUC for referable diabetic retinopathy ranging from 0.889 to 0.983 across 10 externally validated datasets (66). This variability stems from the inclusion of datasets from diverse countries, leading to fluctuations in model performance and introducing uncertainty in clinical applications. Additionally, differences in the quality of fundus color photographs, the algorithms used, and the performance of imaging equipment across studies contribute to significant inconsistencies in AI diagnostic outcomes (67). Notably, these models have been found to be more sensitive to changes in camera equipment than human physicians.

Another critical challenge is the so-called algorithmic “black box” and the resulting clinical trust issues. In AI, “black box models” refer to algorithmic systems—particularly deep learning models—whose internal decision-making processes are difficult to interpret. Although such models often achieve high predictive accuracy, their opacity hampers trust, reproducibility, and clinical adoption. While models like ExplAIn have demonstrated a balance between performance and interpretability for DR diagnosis—through an end-to-end weakly supervised segmentation architecture with generalized occlusion regularization (68)—the issue of transparency remains unresolved. In most cases, interpretability is limited to identifying correlations. For example, Herrero-Tudela et al. employed SHAP to quantify and visualize feature contributions, marking a step forward in model interpretability (69). However, SHAP has inherent limitations: it can indicate the relative importance of metabolites but fails to uncover the causal pathways underlying key pathological mechanisms. This “trustworthy yet uninterpretable” nature continues to pose a major barrier to clinical adoption.

### 6.2 Barriers to real-world applications

The real-world implementation of AI models continues to face substantial challenges. A primary issue is the regulatory divide and the absence of standardized validation protocols. Ong et al. conducted a global analysis of AI as Medical Devices (AIaMDs), identifying 36 devices from 28 manufacturers—97% (35/36) approved in the EU, 22% (8/36) in Australia, and only 8% (3/36) in the United States (70). These findings highlight significant disparities in regulatory approval processes across countries.

Moreover, the false negative and false positive rates of AI models remain critical concerns. A meta-analysis by Wang et al. reported that, despite improvements in AI-based diagnosis of ocular diseases, the false negative rate (FNR) of 12% and false positive rate (FPR) of 8.8% remain non-negligible (71). Even more concerning is the lack of globally harmonized standards for validating AI’s ability to assess dynamic disease progression.

AI deployment is further constrained by resource allocation challenges. Increasing sensitivity may enhance the identification of high-risk patients and improve clinical outcomes, but it also elevates healthcare costs. Conversely, increasing specificity may reduce unnecessary testing but risks missed diagnoses (72). This trade-off places a disproportionate burden on low-income regions. Additionally, AI systems trained on homogeneous datasets may underperform in diverse populations, leading to racial and ethnic disparities in detection rates (73).

Although Vision Transformers (ViTs) have shown promise in detecting diabetic retinopathy in clinical settings, their application is limited by high computational demands. Training ViTs requires high-performance GPUs, with memory usage exceeding 20 GB and power consumption approaching 400 watts per GPU. These resource-intensive requirements pose significant barriers to clinical deployment, particularly in settings with limited infrastructure and funding (74). Collectively, these multifaceted barriers underscore the long and complex path toward real-world adoption of AI in healthcare.

### 6.3 Research gaps and breakthrough directions

Currently, three critical gaps characterize AI research in this field: the absence of cross-model evaluation standards, which leads to fragmentation among diagnostic and therapeutic models—resulting in treatment delays and resource inefficiency; the lack of model generalizability, which impedes large-scale application; and the deficiency of multi-center validation, which causes the performance of otherwise high-precision, single-center models to deteriorate in real-world settings.

To address these challenges, future advancements should focus on three key areas:

1. Establishment of Federated Learning Architecture: Federated learning (FL) offers a promising solution to current limitations. FL enables the development of a unified machine learning model across institutions using decentralized datasets. During training, only model parameters—not raw data—are shared among sites, thereby preserving data privacy. The final model can be retained by a single party or distributed among collaborators (75). This approach facilitates training on larger and more diverse datasets, ultimately enhancing model generalizability.
2. Development of Multimodal Time-Series Models: Chen et al. introduced the MuTri framework to align and transform multimodal data, achieving up to 92% consistency (76). This underscores the feasibility and effectiveness of integrating temporal and multimodal information to improve performance in clinical applications.
3. Promotion of Ultrasound–OCTA–AI Integration: Ultrasound provides a foundational imaging modality capable of overcoming optical occlusion; OCTA offers high-resolution microcirculatory imaging; and AI serves as the integrative engine for fusing heterogeneous data and conducting dynamic risk assessments. The complementary strengths of these three technologies hold promise for mitigating individual limitations and enabling transformative applications in PDR management.
4. Furthermore, effective cost control is essential for the global deployment of AI models. Techniques such as model compression, architectural lightweighting, and hybrid optimization can substantially reduce computational demands. Although these methods may slightly compromise accuracy, they enable broader accessibility and improve overall benefit

## 7 Conclusion

AI has demonstrated substantial value in the diagnosis and treatment of PDR. By enabling efficient analysis of retinal images, guiding surgical procedures with high precision, and optimizing individualized anti-VEGF treatment strategies, AI enhances diagnostic accuracy, therapeutic safety, and procedural efficiency. However, AI-driven PDR management faces challenges, including inadequate data quality, limited model generalizability, the opaque “black-box” nature of algorithms, and unequal distribution of healthcare resources. Addressing these challenges requires overcoming barriers related to data privacy and clinical trust. Proposed solutions include establishing unified regulatory frameworks to enhance system efficacy and safety, developing deep learning systems based on multimodal data fusion, and promoting equitable implementation of automated screening technologies within universal healthcare systems. These measures aim to balance the demands of precision medicine with ethical imperatives for equitable access to care.

**Figure 2:**
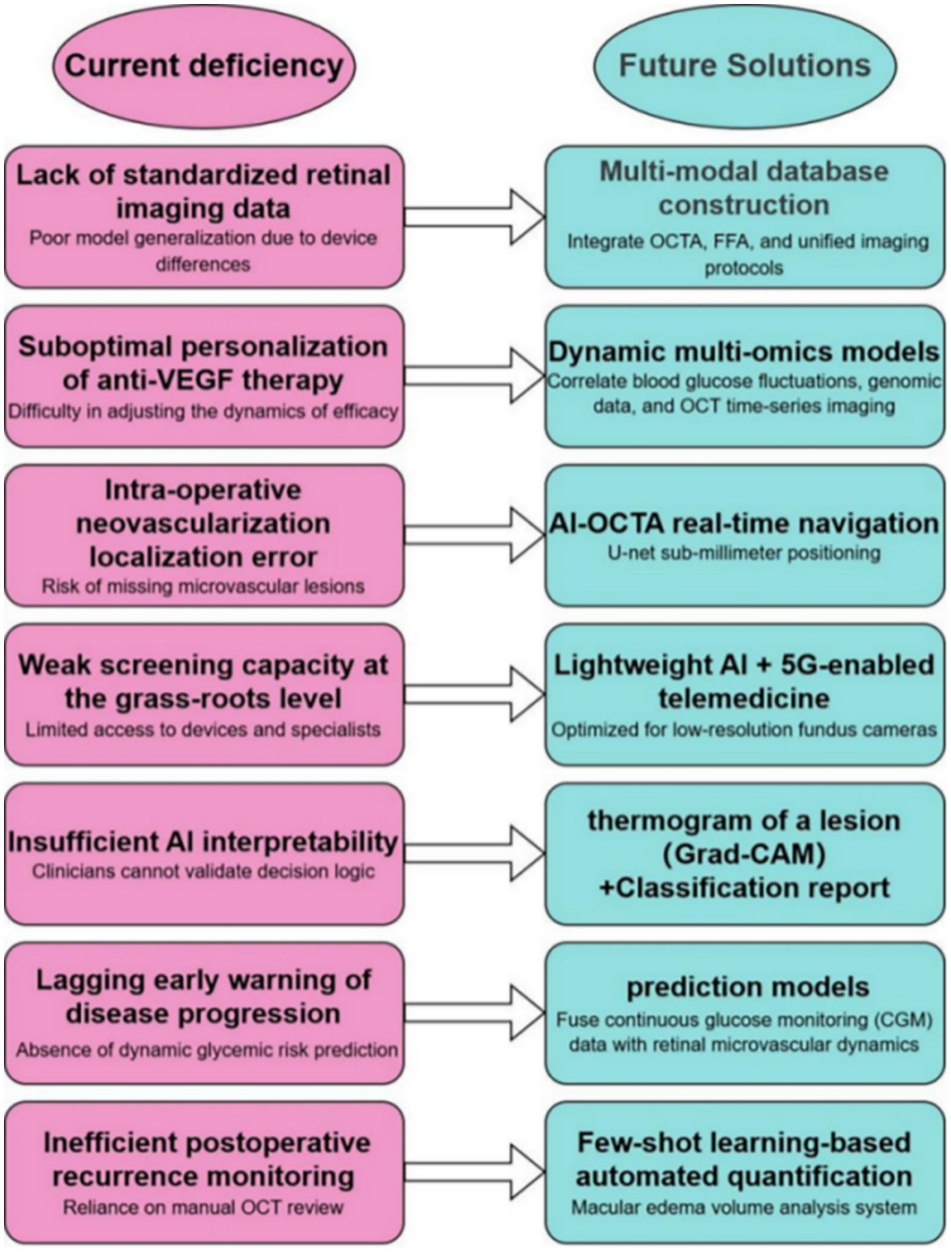
Current challenges and prospects of AI in PDR diagnosis and treatment diagrams illustrating the associations investigated in this study are provided below: Current Challenges (Left Panel): Summarizes key barriers to AI implementation in PDR management, including: Data heterogeneity and lack of standardization. Limited model generalizability across devices/populations. Suboptimal therapy personalization. Technical limitations. Weaknesses in screening capacity and early warning systems. Insufficient AI interpretability. Inefficient postoperative monitoring. Future Solutions and Prospects (Right Panel): Highlights proposed approaches and future goals, such as: Multimodal data integration and database construction. Development of explainable AI frameworks. *Real-time AI-assisted navigation and quantification. Telemedicine solutions leveraging lightweight AI and 5G. * Fusion of continuous monitoring data with retinal imaging. Advanced dynamic modeling. Improved equitable healthcare delivery. Data Sources and References (32, 77–81). OCT, Optical coherence tomography; Grad-CAM, Gradient-weighted Class Activation Mapping; CGM, Continuous Glucose Monitoring System.

In future applications, AI may be utilized to construct time-series forecasting models through multimodal data fusion, enabling end-to-end optimization from early risk prediction to personalized intervention. AI systems could integrate blood glucose fluctuations with real-time retinal microvascular dynamics to predict the risk of vitreous hemorrhage and dynamically adjust anti-VEGF therapy regimens, thereby improving therapeutic response rates while reducing costs. Intelligent decision-support systems may integrate multidimensional data (e.g., genomics, metabolomics) to implement closed-loop care encompassing screening, risk stratification, and treatment optimization. In resource-limited settings, lightweight AI screening devices integrated with 5G-enabled telemedicine networks could help overcome geographic barriers and democratize access to ocular disease prevention and management. In surgical applications, AI-driven intraoperative OCTA navigation systems could precisely localize neovascularization, minimizing the risk of complications. Moreover, by synthesizing genetic and environmental data, AI may support the development of personalized prevention strategies to delay disease progression, while blockchain technology can ensure data security and promote global healthcare equity. Additionally, the integration of advanced ultrasound technologies with AI may provide new opportunities to support intraoperative decision-making. Collectively, these advancements could catalyze a paradigm shift from reactive treatment to proactive health management, particularly benefiting the middle-aged and elderly population (Figure 2).
